# Combination of chemical fingerprint and bioactivity evaluation to explore the antibacterial components of *Salvia miltiorrhizae*

**DOI:** 10.1038/s41598-017-08377-0

**Published:** 2017-08-14

**Authors:** Wei-Jun Kong, Shan-Shan Zhang, Yan-Ling Zhao, Ming-Quan Wu, Ping Chen, Xiao-Ru Wu, Xin-Ping Ma, Wei-Ying Guo, Mei-Hua Yang

**Affiliations:** 1 Institute of Medicinal Plant Development, Chinese Academy of Medical Sciences & Peking Union Medical College, Beijing, 100193 P.R. China; 2 Pharmacy College, Jinzhou Medical University, Jinzhou, 121001 P.R. China; 3 PLA Institute of Chinese Materia Medica, 302 Hospital of People’s Liberation Army, Beijing, 100039 P.R. China; 4 Guizhou Xinbang Pharmaceutical Co. Ltd, Guiyang, 550014 P.R. China; 5 Department of Traditional Chinese Medicine, Air Force General Hospital, PLA, Beijing, 100142 P.R. China

## Abstract

The aim of this study was to explore the possible antibacterial components of *Salvia miltiorrhizae* on *Pseudomonas aeruginosa* using a combination of chemical fingerprint and bioactivity evaluation. The chemical fingerprints of 32 batches of *S*. *miltiorrhizae* samples from different sources were developed using high-performance liquid chromatography with diode array detection, and then were evaluated by similarity analysis and hierarchical clustering analysis. Anti-*P*. *aeruginosa* activity was determined by microcalorimetry. Some crucial thermokinetic parameters obtained from the heat-flow power-time curves of *P*. *aeruginosa* growth in the absence or presence of these *S*. *miltiorrhizae* samples were evaluated using principal component analysis. Thereafter, multiple linear regression analysis was used to analyze the fingerprint-activity relationship between the chemical fingerprints and anti-*P*. *aeruginosa* activity. This established the related equation between the inhibition ratio (*I*, %) of *S*. *miltiorrhizae* samples on *P*. *aeruginosa* and the peak areas of the common peaks. The results showed that the 32*S*. *miltiorrhizae* samples could be grouped into three clusters according to their chemical fingerprints and anti-*P*. *aeruginosa* activities. Protocatechualdehyde, salvianolic acid B, together with three unidentified compounds might be the major components that contributed largely to the antibacterial properties of *S*. *miltiorrhizae* and should be the focus of *S*. *miltiorrhizae* quality control. Thus, this study provided a preferred way for exploring the bioactive components of medicinal plants.

## Introduction

*Salvia miltiorrhizae* (family Lamiaceae), called “Danshen” in Chinese, has been officially listed in the Chinese pharmacopoeia (2015 edition), United States Pharmacopeia (USP39-NF34) and European Pharmacopoeia (EP6.1) as an important and well-known medicinal plant. With pronounced antioxidative, antibacterial and anticoagulant properties, it has been widely used to treat cerebrovascular^1^ and cardiovascular diseases^2^, as well as to prevent inflammation^3^. The main bioactive constituents of *S*. *miltiorrhiza* include phenolic compounds and lipophilic diterpenoid tanshinones, which are typically evaluated during quality control of *S*. *miltiorrhizae* and its related products^4^. Several chromatographic techniques including liquid chromatography (LC)^5–7^, gas chromatography (GC)^8^, high-speed counter-current chromatography (HSCCC)^9^, and thin-layer chromatography (TLC)^10^, have been used to construct chemical fingerprints of *S*. *miltiorrhizae*, which allow researchers to visualize and identify as many components as possible^11^. Among these techniques, LC (used in tandem with various detectors) is typically employed to establish the chemical fingerprint characteristics of *S*. *miltiorrhizae*. *S*. *miltiorrhizae* samples derived from different geographical sources, produced by different manufacturers, or processed by different methods, will have differences in their fingerprints that reflect the differences in their ingredients, as well as their relative contents. Fingerprints have proved to be a useful tool in quality control of *S*. *miltiorrhizae* and related products^12, 13^. However, it is not clear which components are responsible for the pharmacological activities of *S*. *miltiorrhizae* in curing many diseases. Chemical fingerprints alone only improve quality control and standardization, but cannot determine the antibacterial efficacy of *S*. *miltiorrhizae*.

To monitor microbial growth, isothermal microcalorimetry can be used. Isothermal microcalorimetry is a sensitive technique that has been used successfully to evaluate the effects of various substances on microbial metabolism^14^. It provides a continuous measurement of heat-production by variety of microbes in a complex medium, and thereby giving both qualitative and quantitative thermodynamic information on the biological activity of the microbes^15^. By evaluating some crucial thermokinetic parameters such as rate constant and heat-flow power, isothermal microcalorimetry can be used to evaluate the efficacy of other substances on the microbes^16^. This method only requires knowledge of the initial and final energetic states of the system, and is independent of the organisms studied or reaction pathways involved^17^. As a powerful and appealing tool, isothermal microcalorimetry has been used to describe the effects of many medicinal plants on various types of microbes^18–20^.

One of the microbes is the *Pseudomonas aeruginosa*. It is a ubiquitous, non-fermentative gram-negative microbe^21^ that causes many kinds of human infections^22^, such as bacteraemia in burn victims, urinary-tract infections in catheterized patients, and hospital-acquired pneumonia in patients on respirators^23^. These infections are difficult to eradicate, in part because of the natural resistance of the bacterium to antibiotics, and can ultimately lead to pulmonary failure and death^24^. Therefore, it is necessary to screen for highly effective anti-*P*. *aeruginosa* compounds with low toxicity using appropriate techniques. Recently, the antibacterial effects of *S*. *miltiorrhizae* have attracted growing attention. Nevertheless, to date, no research has focused on identifying the major constituents of *S*. *miltiorrhizae* that mainly contribute to its anti-*P*. *aeruginosa* activity.

Therefore, this study combined the chemical fingerprints and anti-*P*. *aeruginosa* effects of *S*. *miltiorrhizae* to establish an integrated evaluation system for exploring the putative active components of this medicinal plant representing its biothermal activity. First, the chemical fingerprints were developed from 5% methanol extracts of 32 batches of *S*. *miltiorrhizae* from different sources by high-performance liquid chromatography with diode array detection (HPLC-DAD). This allowed us to characterize as many components as possible, and the peak area of each component was recorded. Then, the anti-*P*. *aeruginosa* activity of each *S*. *miltiorrhizae* sample was determined by microcalorimetry. Specifically, some crucial thermokinetic parameters obtained from the heat-flow power-time curves of *P*. *aeruginosa* growth in the absence and presence of each *S*. *miltiorrhizae* sample were evaluated. The chemical fingerprints and biothermal activities of *S*. *miltiorrhizae* from different sources were combined to establish fingerprint-activity relationships with the help of chemometrics by carrying out similarity analysis, principal component analysis and multiple linear regression analysis. Understanding these relationships allowed us to further explore the active components of *S*. *miltiorrhizae* that contributed largely to the anti-*P*. *aeruginosa* activity. The approach described in this study provides a preferred model for future studies. It illustrates how chemical fingerprints can be combined using LC method and biothermal activity by introducing microcalorimetry technique to study the active components of medicinal plants and other valuable materials.

## Results and Discussion

### HPLC chemical fingerprints

#### Method validation

The results showed that the relative standard deviation (RSD) values of peak areas for evaluating the precision, repeatability and stability of the method were less than 8.79%, 8.28% and 6.82% respectively. This indicates that the HPLC method was precise and sensitive enough for the qualitative analysis and the establishment of HPLC fingerprints. Thus, the results have proved satisfactory stability and reproducibility of the sample solution.

#### HPLC fingerprinting and similarity analysis

**Figure 1:**
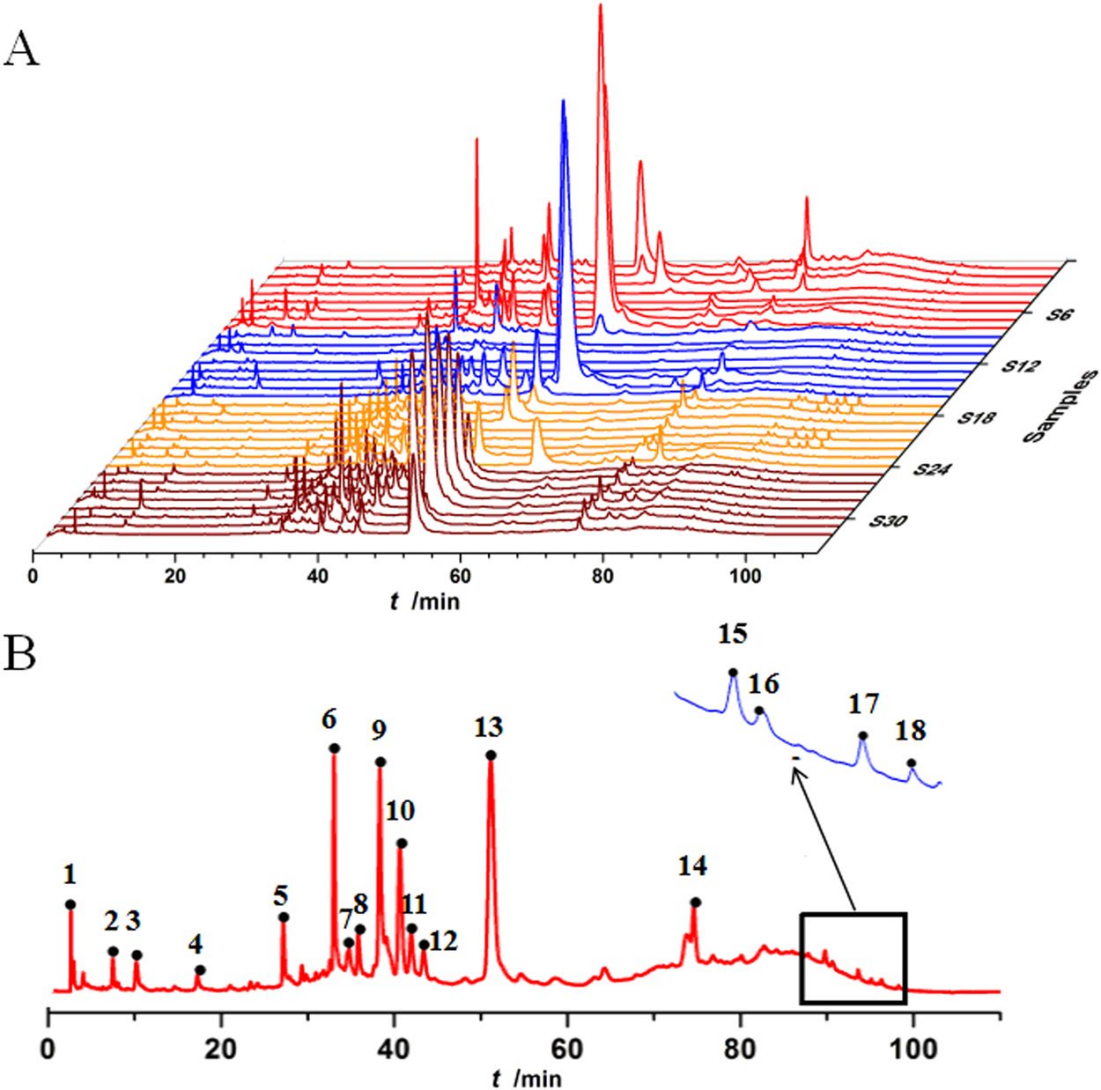
Typical HPLC-DAD fingerprints of 5% methanol extracts of 32 batches of *S*. *Miltiorrhizae* from different sources.

Chemical fingerprints are strongly recommended by many international organizations, including Chinese materia medicas, to accurately profile integrities of complicated matrices for the authentication and identification of their components. The evidence described above shows that the validated HPLC-DAD method was valid and suitable for stable and reproducible chemical fingerprints of *S*. *miltiorrhizae* samples. Solutions of 32 batches of *S*. *miltiorrhizae* samples collected from different sources were prepared under the optimum chromatographic conditions (see Materials and Methods) and loaded into the HPLC system. Typical HPLC fingerprints were recorded (Fig. 1A), indicating that these samples had similar chemical profiles with some common chemical components. A reference fingerprint was generated by comparing samples’ DAD spectra and HPLC retention times (Fig. 1B). This reference fingerprint was created with the assumption that all the common peaks should be observed in every sample. Eighteen common characteristic peaks were identified in the reference chromatogram. Table S1 listed the average peak area and relative retention time of the 18 common characteristic peaks in the 32 samples.

Although all samples shared similar HPLC fingerprints in Fig. 1A, the average peak areas of the 18 common peaks varied between samples. The coefficients of variance (C.V.%) for peak area ranged from 65.9–178.0%. This indicated that, in addition to qualitative differences between samples, there were significant differences in the amount of each component between samples.

Next, the professional similarity evaluation software was used to perform similarity analysis on the data reported in Table S1. The similarities between the reference fingerprint and each chromatographic profile of the 32 *S*. *miltiorrhizae* samples were evaluated by calculating the correlation coefficients, which for the 32 samples were as follows: 0.876, 0.872, 0.808, 0.856, 0.824, 0.867, 0.962, 0.962, 0.963, 0.385, 0.445, 0.302, 0.447, 0.96,0 0.966, 0.844, 0.919, 0.881, 0.935, 0.96, 0.966, 0.982, 0.953, 0.967, 0.963, 0.921, 0.877, 0.820, 0.797, 0.933, 0.961, and 0.922. Significant variations of correlation coefficients ranging from 0.302 to 0.982 further indicated that the chemical fingerprints and internal qualities of *S*. *miltiorrhizae* samples vary greatly between samples derived from different sources.

#### Hierarchical Clustering Analysis

**Figure 2:**
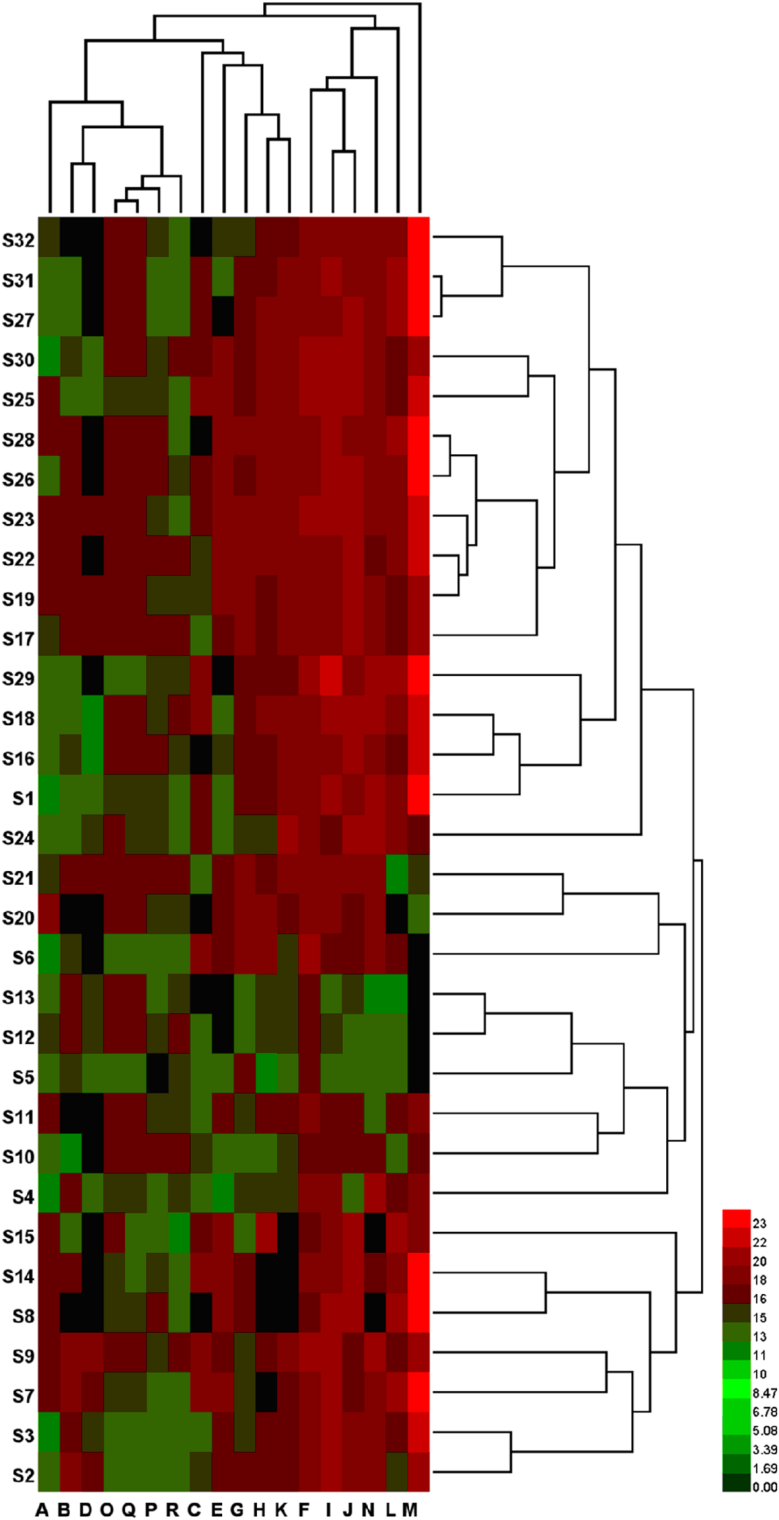
Hierarchical clustering analysis of *S*. *miltiorrhizae* samples. Samples S1–S32 are performed using HemI statistics software (Heatmap Illustrator, Version 1.0). The between-groups linkage method as the amalgamation rule and the squared Euclidean distance were selected to establish clusters.

In order to explore the natural clustering of *S*. *miltiorrhizae* samples from different sources, hierarchical clustering analysis (HCA) was performed on HPLC fingerprints. Thirty-two samples were analyzed using HCA of the peak areas of the 18 common analytical markers in the HPLC fingerprints. The between-groups linkage method was chosen as average linkage and the squared Euclidean distance was selected to establish clusters, and to assess the resemblance of the 32 batches of samples. The resulting hierarchical cluster heat-map in Fig. 2 produced well-defined clusters and showed the peak area profile of the 18 common peaks. Samples were obviously grouped into three main clusters. Samples S2 and S3 collected from Sichuan province, S7–S9 from Henan province, and S14 and, S15 from Beijing city were categorized into cluster I; Samples S4–S6 collected from Sichuan province, S10–S13 from Shandong province, and S20 and S21 from Guizhou province were grouped into a second distinct cluster (cluster II). The remaining samples including S16–S19, S22–S32 from Guizhou province and S1 from Sichuan province were put into cluster III. As shown in Fig. 2, samples from the same source were typically grouped together. The distances between samples in the same cluster were shorter than distances between samples not in the same cluster, indicating that HPLC chemical fingerprints and internal qualities were more similar within clusters compared to samples in other clusters. The distance between cluster II and III was shorter than the distances between these clusters and cluster I. This result could be explained by the fact that samples were collected different geographic settings, which have different cultivated climates, soil quality, longitude and latitude. These results agreed with the findings from the similarity analysis, and with a simple visual comparison of HPLC chromatograms, including comparison of color variation between the 18 common peaks (Fig. 1A), which might reflect their different bioactivity. Therefore, in the next section, the biothermal activities of the 32 samples on *P*. *aeruginosa* growth were studied.

### Biothermal activity evaluation

#### Metabolic power-time curves of P. aeruginosa growth

**Figure 3:**
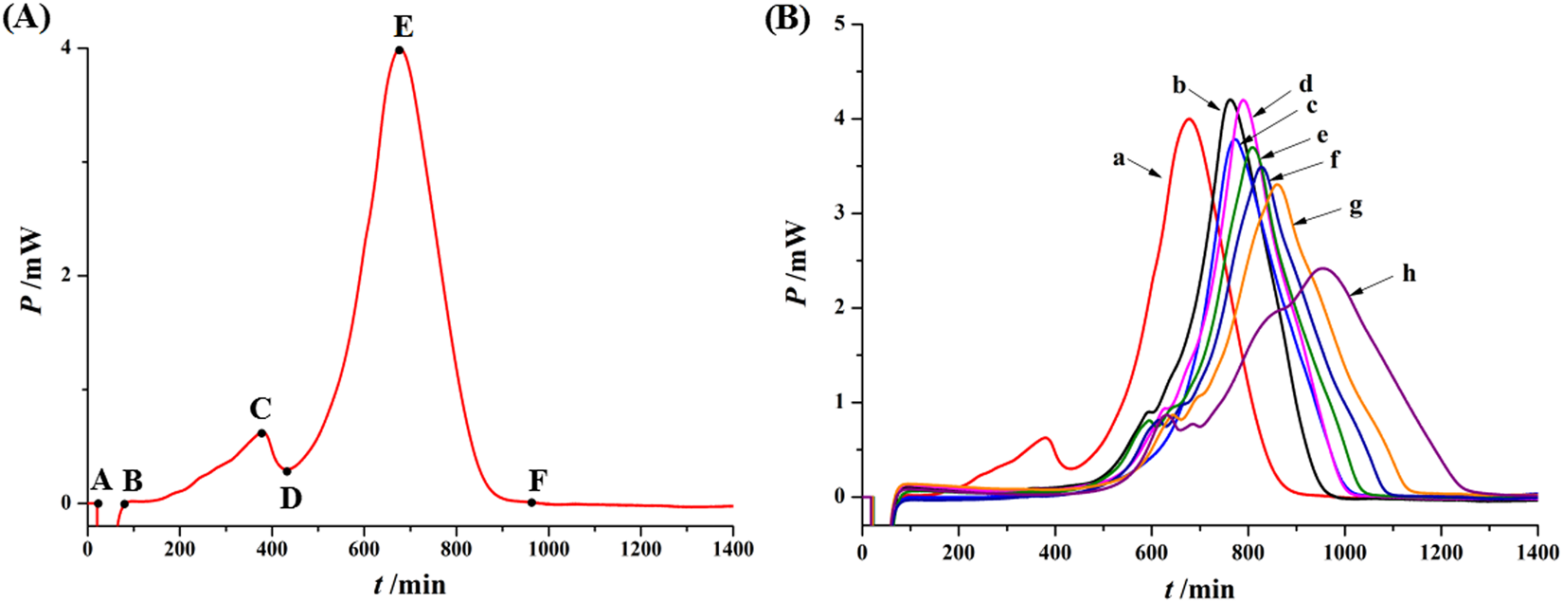
The power-time curves of *P*. *aeruginosa* growth at 37 °C (**A**) without the samples, and (**B**) affected with different concentrations of *S*. *miltiorrhizae* extracts. a–h Represent the concentrations of 0, 50, 100, 250, 500, 750, 1000 and 2000 μg/mL of *S*. *miltiorrhizae* extracts.

Using the microcalorimeter, the normal power-time curve of *P*. *aeruginosa* growth at 37 °C without any substance was recorded and shown in Fig. 3A. Because *P*. *aeruginosa* was grown under airtight conditions in the glass ampoules, bacterial consumption of nutrients and oxygen was limited. Therefore, the growth curve of *P*. *aeruginosa* was divided into five phases: lag phase (A-B), first exponential phase (B-C), transition phase (C-D), second exponential phase (D-E), and decline phase (E-F).

#### Growth rate constant (k) of P. aeruginosa

The heat-flow power-time curves of *P*. *aeruginosa* growth in the LB culture medium at 37 °C monitored by the microcalorimeter showed that the bacteria growth can be described by an exponential model as follows:

1 $${P}_{t}={P}_{0}\,\exp (kt)\quad {\rm{or}}\quad \mathrm{ln}\,{P}_{t}=\,\mathrm{ln}\,{P}_{0}+kt$$

where *P*_0_ and *P*_t_ represent the heat-flow power at time 0 and *t*, respectively. The exponential phase of *P*. *aeruginosa* growth corresponded to Eq. (1). Using this equation, the growth rate constants (*k*) including *k*_1_ and *k*_2_, which corresponded to the first and second exponential phase of *P*. *aeruginosa* growth, could be calculated by fitting ln *P*_t_ and *t* to a linear equation. The values of *k*_1_ and *k*_2_ in the absence of other substances of three reduplicative experiments were *k*_1_ = (0.00850 ± 0.00134) min^−1^ and *k*_2_ = (0.01468 ± 0.00246) min^−1^, with correlation coefficients greater than 0.99284, indicating good reproducibility of the microcalorimetry measurements.

#### Biothermal activity of S. miltiorrhizae on P. aeruginosa

Addition of different concentrations of *S*. *miltiorrhizae* sample solution to the internal system of *P*. *aeruginosa* growth in the glass ampoule would influence bacterial growth. As shown in Fig. 3(B), addition of 1000 µg/mL of *S*. *miltiorrhizae* sample solution produced a strong inhibitory effect on *P*. *aeruginosa* growth by expressing satisfactory power-time curve, as well appropriate peak height and appearance time of the first and second highest peaks. Therefore, a final concentration of 1000 µg/mL was used for all *S*. *miltiorrhizae* sample solutions added to the ampoule.

**Figure 4:**
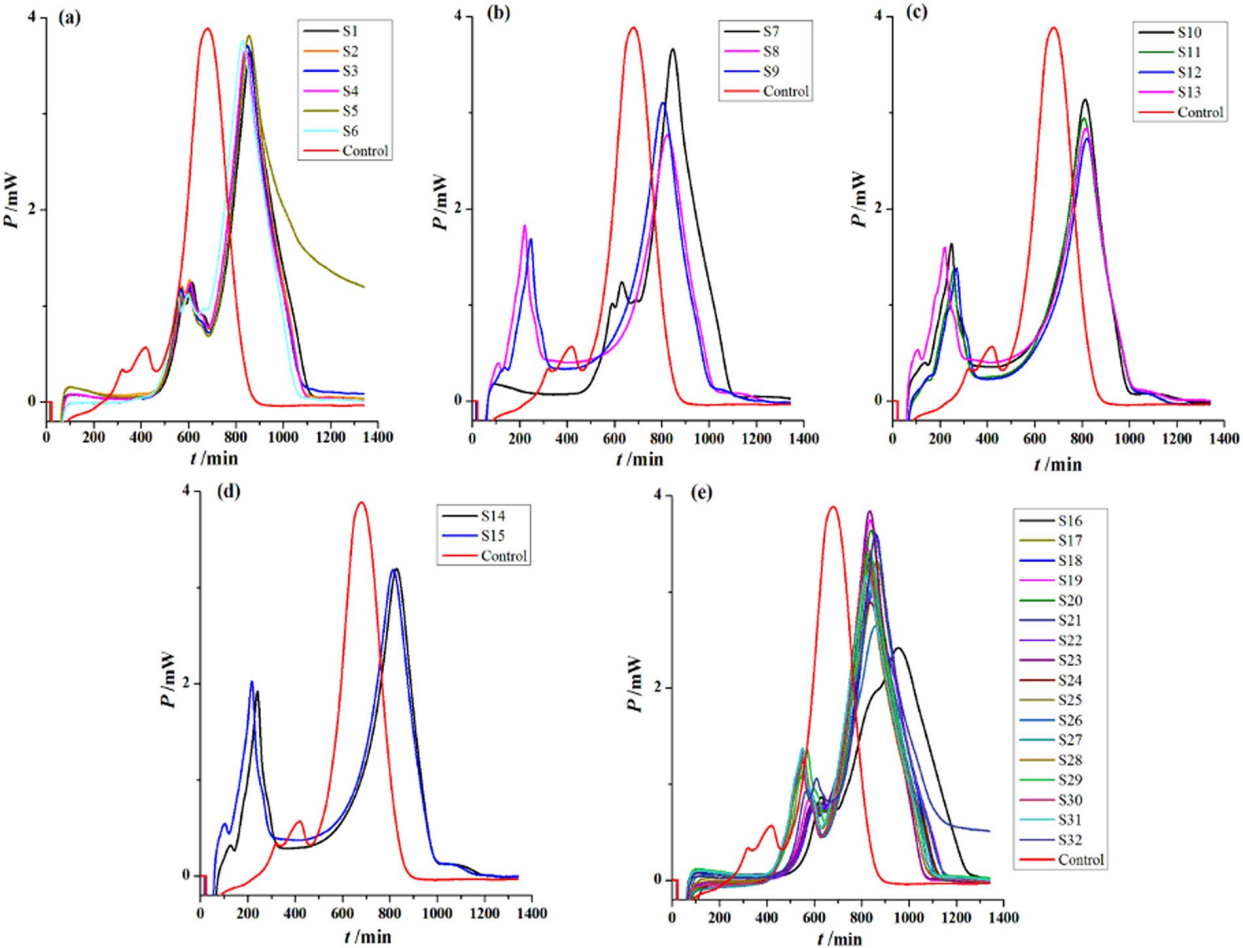
The power-time curves of *P*. *aeruginosa* growth at 37 °C affected by *S*. *miltiorrhizae* extracts from different sources. Samples are from (**a**) Sichuan province, (**b**) Henan province, (**c**) Shandong province, (**d**) Beijing Tongrentang and Hongshengtang Co. Ltd., and (**e**) Guizhou Xinbang Pharmaceutical Co. Ltd.

The heat-flow power-time curves of *P*. *aeruginosa* growth at 37 °C in the presence of 32 *S*. *miltiorrhizae* sample solutions are shown in Fig. 4. It was found that the shape of these power-time curves was similar to the control without *S*. *miltiorrhizae* sample, i.e., the five phases of the curves still existed; however, the first and second peaks’ height of the treatment sample was lower and the appearance time was prolonged relative to control, illustrating that the 32 *S*. *miltiorrhizae* samples all had inhibitory effects on *P*. *aeruginosa* growth. The samples’ anti-*P*. *aeruginosa* activity could also be quantitatively evaluated by examining the changes in some thermokinetic parameters (Table 1) obtained from the power-time curves. These parameters included the growth rate constants *k*_1_ and *k*_2_ (min^−1^) for the first and second exponential phase, the two maximal heat-flow powers *P* ^1^_m_ and *P* ^2^_m_ (mW), the appearance time *t* ^1^_m_ and *t* ^2^_m_ (min) of the two highest peaks, and the heat outputs *Q*_1_ and *Q*_2_ for the two growth stages associated with the total heat output *Q*_t_.

**Table 1:** Thermokinetic parameters obtained from the power-time curves of *P*. *aeruginosa* growth in the presence of 1000 μg/mL of *S*. *Miltiorrhizae* from different sources.

Samples	*k*_1_ (min^−1^)	*k*_2_ (min^−1^)	*t* ^1^_m_ (min)	*t* ^2^_m_ (min)	*P* ^1^_m_ (mW)	*P* ^2^_m_ (mW)	*Q*_1_ (J)	*Q*_2_ (J)	*Q*_t_ (J)	*I* (%)
Control	0.01158	0.01576	419.3	678.8	0.5693	3.8873	10.68	45.33	56.00	0
S1	0.02315	0.01187	614.8	861.0	1.2458	3.6408	11.40	45.53	56.93	24.68
S2	0.02275	0.01215	604.3	855.7	1.2719	3.8157	10.52	44.43	54.95	22.91
S3	0.02721	0.01281	601.3	848.3	1.192	3.7101	10.37	44.26	54.63	18.72
S4	0.02162	0.01223	608.3	842.5	1.2412	3.6648	10.11	70.90	81.02	22.40
S5	0.02527	0.01265	603.3	855.8	1.1363	3.8091	8.21	47.84	56.05	19.73
S6	0.0188	0.01146	597.3	828.0	1.1198	3.7552	8.94	46.81	55.75	27.28
S7	0.0208	0.01141	631.2	845.8	1.2427	3.6652	13.53	42.03	55.55	27.60
S8	0.01867	0.00859	221.7	822.7	1.831	2.7699	12.22	44.44	56.65	45.49
S9	001554	0.00717	247.5	803.7	1.6909	3.1044	12.77	43.36	56.12	54.51
S10	0.01548	0.00742	248.5	813.8	1.6392	3.1408	8.85	41.53	50.38	52.92
S11	0.02002	0.00848	255.3	807.5	1.358	2.9446	9.73	37.78	47.51	46.19
S12	0.02489	0.00728	269.0	820.2	1.3849	2.7312	13.90	42.29	56.19	53.81
S13	0.01698	0.00536	219.8	815.3	1.6036	2.8399	11.52	43.37	54.89	65.99
S14	0.02739	0.00623	241.0	827.3	1.924	3.1977	14.99	43.95	58.94	60.47
S15	0.01672	0.00585	217.5	813.8	2.0266	3.189	4.73	49.72	54.45	62.88
S16	0.01608	0.00791	630.7	951.7	0.8697	2.4192	4.36	47.89	52.25	49.81
S17	0.02112	0.00984	592.3	864.3	0.7807	3.3247	4.03	49.44	53.47	37.56
S18	0.02153	0.01064	602.2	860.7	0.7672	3.6019	3.85	46.81	50.66	32.49
S19	0.02252	0.0112	578.8	836.0	0.8518	3.7503	3.56	48.42	51.97	28.93
S20	0.02288	0.01105	593.3	839.7	0.7637	3.6396	4.57	48.15	52.72	29.89
S21	0.02028	0.01095	595.2	836.7	0.8176	3.3659	4.03	48.84	52.87	30.52
S22	0.02271	0.01064	599.8	849.8	0.8279	2.9895	4.57	48.11	52.69	32.49
S23	0.01406	0.00928	597.0	833.5	0.8005	3.8425	8.46	43.67	52.13	41.12
S24	0.02119	0.00911	551.5	839.2	1.3449	2.8918	8.70	42.98	51.68	42.20
S25	0.02316	0.01036	547.3	834.7	1.3404	3.2771	9.06	42.46	51.52	34.26
S26	0.02232	0.01093	556.8	859.7	1.3062	2.6475	8.25	44.11	52.36	30.65
S27	0.02108	0.00907	549.2	843.3	1.3135	3.0236	8.81	43.97	52.79	42.45
S28	0.0203	0.0094	557.0	818.2	1.3233	3.3414	9.99	44.91	54.90	40.36
S29	0.01915	0.0116	570.7	834.5	1.3612	3.415	8.45	45.11	53.57	26.40
S30	0.02135	0.01152	546.8	823.0	1.3453	3.4572	9.72	45.12	54.83	26.90
S31	0.0193	0.01136	550.7	823.0	1.3766	3.1628	8.68	55.25	63.93	27.92
S32	0.02592	0.0109	609.5	857.2	1.0619	3.5857	10.68	45.33	56.00	30.84

The thermokinetic parameters reported in Table 1 were different for all the samples. In general, the smaller the values of *k*_1_, *k*_2_, *P* ^1^_m_, *P* ^2^_m_ are, and the bigger the values of *t* ^1^_m_, *t* ^2^_m_ are, the stronger anti-bacterial activity a substance possesses^25^. From Table 1 and Figure S1, it could be figured out that the nine parameters exhibited different increasing or decreasing trends to various degrees. Thus, examination of a single parameter was not helpful for accurate assessment of the biothermal activities of the 32 *S*. *miltiorrhizae* samples. Therefore, it was necessary to identify the most important parameter(s) for quick and accurate assessment. To solve this problem, PCA was introduced.

#### Results of PCA

**Figure 5:**
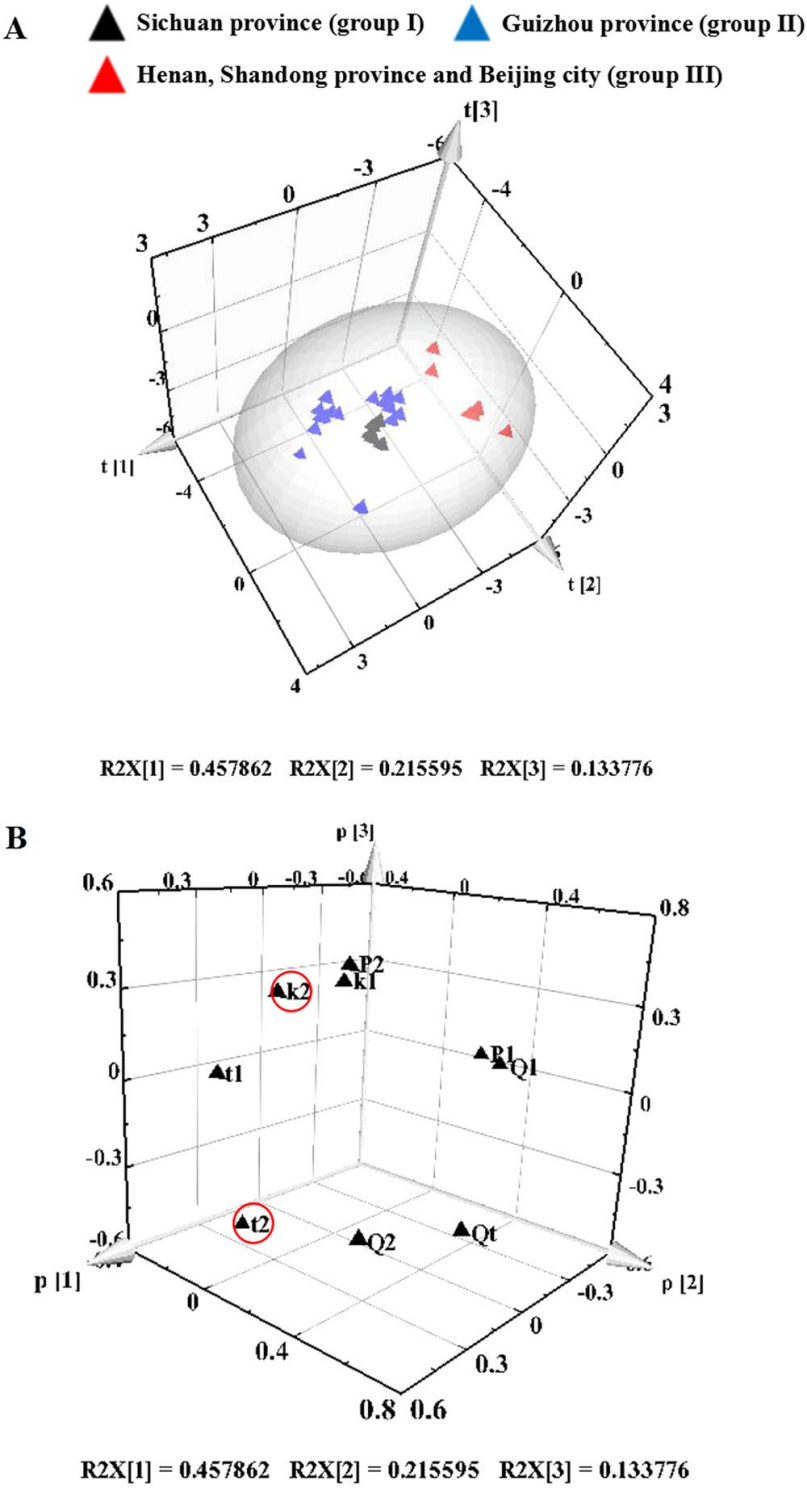
(**A**) The score plot of PCA (*t*1/*t*2/*t*3); S1–S32 represent the *S*. *miltiorrhizae* samples. (**B**) The loading plot of PCA (*p*1/*p*2/*p*3). The main parameters are marked with a circle.

PCA can summarize and contract the information residing in the multi-dimensional original data, i.e., either the chemical descriptors, the biological responses, or both, into a few descriptive dimensions that are denoted as principal components to represent the main variation in the data^26^. Therefore, PCA is recommended to reduce the computation burden. Here, the values of the nine thermokinetic parameters in Table 1 were set as variables, and the 32 samples were set as observations. Then, the variables were centered and scaled to “Unit Variance” before performing the PCA by SIMCA-P. The results in Fig. 5 show that the first three principal components (*t*1/*t*2/*t*3) explained 80.7% of the total variance. Moreover, the 32 *S*. *miltiorrhizae* samples were divided into three groups in Fig. 5A: group I included samples S1–S6 from Sichuan province, group II contained samples S16–S32 from Guizhou province, and group III contained samples S7–S15 from Henan and Shandong provinces, and Beijing city. The samples of interest were clearly grouped into distinct classes of anti-*P*. *aeruginosa* activity according to their collection site. This might be due to the differences in harvesting time, longitude and latitude of the growth site, or differences in climate, soil and water conditions, and other factors. The results obtained from PCA based on the anti-*P*. *aeruginosa* activities of *S*. *miltiorrhizae* samples were in accordance with that of HCA of the chemical fingerprints.

Additionally, the 3D loading plots (*p*1/*p*2/*p*3 loading plot in Fig. 5B) indicate that *t* ^2^_m_ and *k*_2_ were the two most important thermokinetic parameters that could be used to access the anti-*P*. *aeruginosa* activity of *S*. *miltiorrhizae* samples. These two parameters could be used to calculate an inhibition ratio to quantitatively compare the biothermal activities between samples.

### Inhibition ratio (*I*, %) of *S. miltiorrhizae* samples on *P. aeruginosa*

To quantitatively describe the inhibitory effects of *S*. *miltiorrhizae* samples on *P*. *aeruginosa*, the inhibition ratio (*I*, %) is calculated according to the following formula:

2 $$I=[({k}_{(2,0)}-{k}_{(2,s)})/{k}_{(2,0)}]\times 100 \% $$

where *k*_(2,0)_ is the growth rate constant for the second exponential growth phase of *P*. *aeruginosa* in the absence of any substance (the control) and *k*_(2_,_s)_ is the growth rate constant in the presence of an *S*. *miltiorrhizae* sample. *I* reflects the inhibition extent and effects of *S*. *miltiorrhizae* on the bacteria’s growth. Higher the values of *I* represent stronger the inhibitory effects of *S*. *miltiorrhizae* on *P*. *aeruginosa* growth.

**Figure 6:**
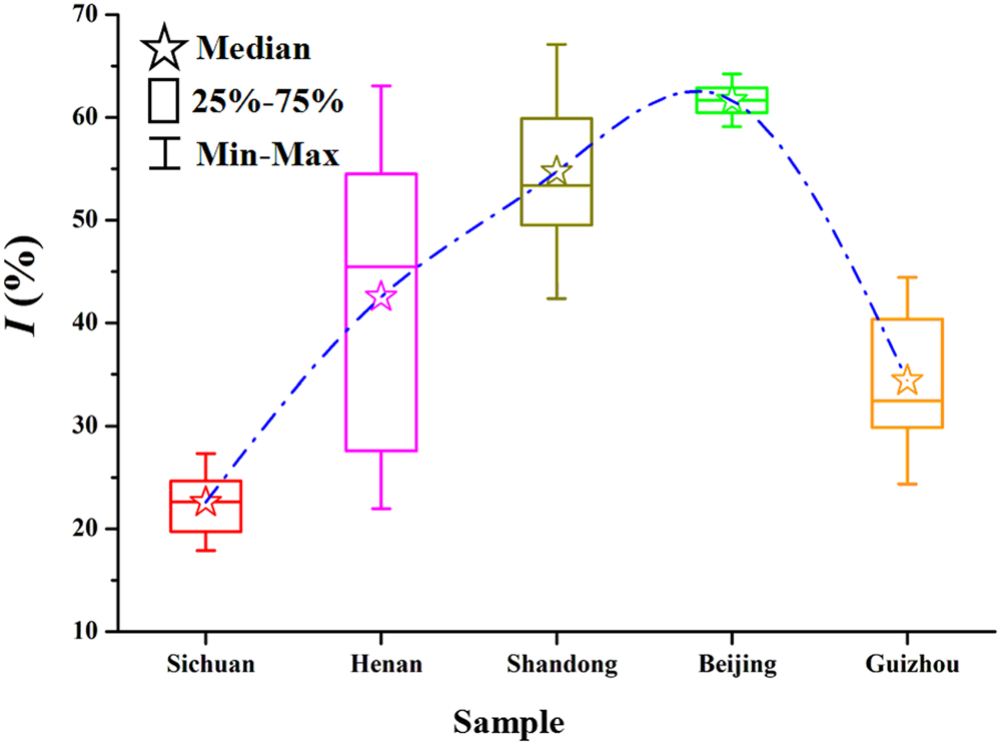
Box and Whisker plots of the inhibition ratio (*I*, %). Origin 8.5 software was used to generate these plots and show the minimum and maximum value to indicate the range, the median, 25% and 75% quartile.

Based on the parameter *k*_2_ in Table 1, *I* values were calculated according to Eq. (2) (Table 1). The maximum value of *I* is 65.99% for sample S13 collected from Shandong province, which is almost four times greater than the minimum value of *I* (18.72%) for sample S3 from Sichuan province. These results further illustrate the substantial differences in the anti-*P*. *aeruginosa* activity of the 32 *S*. *miltiorrhizae* samples from various sources. Box and whisker plots in Fig. 6 show the markedly wide distribution of *I* values, as well as the changing trends, for all samples. The minimum and maximum values (which indicate the range of *I*), the median value (which represent the average), and the 25% and 75% quartile values of *I* (depicted as a box to indicate the variability around this average) demonstrated that samples collected from Beijing city had the greatest inhibitory effect, followed by samples from Shandong, Henan, Guizhou and Sichuan provinces. Taking the trending line to show the median values into account, it can be concluded that the *S*. *miltiorrhizae* samples from Beijing city exhibited the strongest anti-*P*. *aeruginosa* activities, while the samples from Sichuan province had the smallest inhibitory effects. Combining these findings with the groupings of samples in Fig. 5A, we further predicted that the magnitude of the three groups’ anti-*P*. *aeruginosa* activities should be ordered as follows: group III > group II > group I. Furthermore, within group III, the order of greatest inhibitory effect would be: Beijing city > Shandong province > Henan province. Different harvesting time, longitudes and latitudes location of growth sites, and different climates, soil and water conditions might account for the different anti-*P*. *aeruginosa* activities between samples, which should be explored in detail in future studies.

### Results of MLRA

The results described above demonstrated that *S*. *miltiorrhizae* samples from various sources had different chemical profiles and different anti-*P*. *aeruginosa* activities. However, which of the main active components within the chemical profile contributed largely to the anti-*P*. *aeruginosa* activities of *S*. *miltiorrhizae* samples were not clear. Therefore, MLRA was used to help in establishing the fingerprint-activity relationships by comparing chemical fingerprints to biothermal activity.

MLRA generalizes directly to multiple predictor variables and the multiple linear regression equation relates the continuous response variables (*Y*) to predictor variables (*x*). In this study, it was found that a strong linear relationship exists between $\sqrt{I}$ (the square root of *I*/%) and the normalized peak area values. Therefore, the value of $\sqrt{I}$ in Table 1 was set as the dependent variable (*Y*) and the normalized peak area values of 18 chromatographic peaks (described in Table S1) were set as independent variables *x*_1_, *x*_2_, *x*_3_, … *x*_18_ by fitting a linear equation with multiple linear regression model. The results showed that the *F* value was 1.658 and the corresponding *P* value was 0.000 (ie., less than 0.05), demonstrating that the MLRA model was satisfactory. The regression equation was listed as:

3 $$\begin{array}{rcl}Y & = & -8.918\times {10}^{-17}+0.212{x}_{1}-0.096{x}_{2}+0.329{x}_{3}+0.535{x}_{4}-0.391{x}_{5}+0.12{x}_{6}\\  &  & -0.234{x}_{7}+0.483{x}_{8}-0.150{x}_{9}-0.055{x}_{10}-0.141{x}_{11}-0.183{x}_{12}+0.603{x}_{13}\\  &  & -0.143{x}_{14}+0.270{x}_{15}+0.124{x}_{16}+0.506{x}_{17}-0.663{x}_{18}\end{array}$$

The equation above describes the degree to which the 18 peaks or components in *S*. *miltiorrhizae* samples contributed to the anti-*P*. *aeruginosa* activity. *x*_4_, *x*_8_, *x*_13_, *x*_17_ and *x*_18_ had the greatest important influence on *I*, with correlation coefficients >0.4, indicating that peak 4 (protocatechualdehyde), peak 8 (unidentified compound), peak 13 (salvianolic acid B), peak 17 (unidentified compound), and peak 18 (unidentified compound) might be the main bioactive components that conferred the anti-*P*. *aeruginosa* activity of *S*. *miltiorrhizae* samples. In contrast, other peaks, including peak 2 (danshensu), peak 6 (protocatechuic acid), and peak 10 (rosmarinic acid) contributed little to the anti-*P*. *aeruginosa* activity. Among the five components with major contributions to activity, peaks 4, 8, 13, and 17 were positively correlated to *I*, which meant that concentrations of these four compounds were associated with greater anti-*P*. *aeruginosa* activity. Peak 18, however, was negatively correlated with antibacterial activity; therefore increased concentration of this compound would decrease antibacterial activity. Interestingly, Fig. 1B and Table S1 show that the areas of peak 4 (protocatechualdehyde), peak 8 (unidentified compound), peak 17 (unidentified compound) and peak 18 (unidentified compound) are often quite small, in spite of these components’ major influence on anti-*P*. *aeruginosa* activity. In contrast, peak 13 (salvianolic acid B), together with other peaks or components that have bigger peak areas (therefore higher contents) exhibited minor influences on anti-*P*. *aeruginosa* activity. In other words, the most abundant compounds in *S*. *miltiorrhizae* might not be the major contributors to antibacterial activity, and some compounds (including unidentified compounds) should be the focus of future studies. The chemical structures of compounds 8, 17, and 18 need be identified.

## Conclusion

To explore the main antibacterial components of *Salvia miltiorrhizae* that inhibit the growth of *P*. *aeruginosa*, this study established the chemical fingerprints of 32 batches of *S*. *miltiorrhizae* samples from different sources using HPLC-DAD method. Further, the biothermal activities of these samples on *P*. *aeruginosa* growth were determined using microcalorimetry. With the help of chemometric approaches such as HCA, PCA and MLRA, the chemical fingerprints and biothermal activity data were evaluated together to establish the fingerprint-activity relationships. These approaches gave similar results. The 32 *S*. *miltiorrhizae* samples were grouped into three clusters according to their chemical fingerprints and anti-*P*. *aeruginosa* activities. All the samples from various sources expressed strong anti-*P*. *aeruginosa* activities. Furthermore, protocatechualdehyde, salvianolic acid B and three unidentified compounds might be the major components that primarily contribute to antibacterial activity, and could be important factors for quality control of *S*. *miltiorrhizae*.

To the best of our knowledge, this is the first report to establish fingerprint-activity relationship for exploring the possible antibacterial components of *S*. *miltiorrhizae*. This study’s approach could act as a model for future studies. We believe that our method will provide a powerful way to combine chemical fingerprints and biothermal activity evaluation to identify the bioactive components in medicinal plants and other valuable materials, which could be crucial for quality control.

## Materials and Methods

### Materials

Six top-geoherbs of *S*. *miltiorrhizae* samples (labeled as S1–S6) and the rest of 3 batches (S7–S9), 4 batches (S10–S13), 2 batches (S14 and S15) were purchased from Sichuan province, Henan province, Shandong province, Tongrentang and Hongshengtang Co. Ltd., respectively, the other 17 batches of *S*. *miltiorrhizae* samples (labeled as S16–S32) were offered by Guizhou Xinbang Pharmaceutical Co. Ltd., which were authenticated by Prof. Yulin Lin (Institute of Medicinal Plant Development, Chinese Academy of Medical Sciences and Peking Union Medical College, Beijing, China). All samples were collected in sterilized polyethylene bags and stored at −20 °C before analysis.

The standards of salvianolic acid B, danshensu, protocatechuate, protocatechualdehyde and rosmarinic acid (purity >99%) were provided by Chengdu Must Bio-Technology Co. Ltd, Chengdu, China.

*Pseudomonas aeruginosa* (ATCC27853) was provided by Clinical Examination Center of 302 Hospital of People’s Liberation Army, Beijing, China. The Luria-Bertani (LB) culture medium was prepared by dissolving 10 g of tryptone, 5 g of yeast extract, and 5 g of NaCl in 1000 mL of distilled water at pH7.0–7.2. Then, the LB culture medium was sterilized by autoclaving at 0.1 MPa and 121 °C for 30 min. Initially, *P*. *aeruginosa* was inoculated in a 30-mL sterilized flask and incubated in the shaker at rotation rate of 110 rpm for 6 h at 37 °C, then was stored in a refrigerator at 4 °C.

Methanol and acetonitrile (HPLC grade) were obtained from Fisher Scientific (Fair Lawn, NJ, USA). Formic acid was bought from Xilong Chemical Co., Ltd. (Guangdong, China). Purified water was used (Wahaha, Hangzhou, China). A KQ-500 ultra-sonic cleaning bath (50 × 30 × 35 cm) manufactured by Kunshan Ultrasonic Instrument Co., Ltd. was used (Jiangsu, China).

### Instrumentation

HPLC fingerprints were measured with a Shimadzu Prominence system equipped with LC-20AT quaternary gradient pump, a SPD-M20A diode array detector (DAD), CBM-20A communication bus module, CTO-20A thermostatic column compartment, SIL-20A autosampler and Shimadzu LC solution software (Version 1.21 SP1).

A 3114/3236 TAM air microcalorimeter (Thermometric AB, Sweden) was used to determine the metabolic heat-flow power-time curves of *P*. *aeruginosa* growing in the LB culture medium. As an isothermal heat conduction calorimeter operating in the microwatt range, the microcalorimeter has eight calorimetric channels to keep the temperature in the range of 20–80 °C. All channels were mounted together to form a single heat-sink block housed in a temperature-controlled air thermostat. Each calorimetric channel was constructed in twin configuration with one side for the sample and the other side for a static reference. For more details about the instrument, refer to ref. 27.

### HPLC fingerprints

#### HPLC conditions

For chromatographic separation, a COSMOSIL Packed 5C18-PAQ column (5 μm, 250 mm × 4.6 mm, COSMOSIL, Japan) was used at 30 °C. The mobile phase consisted of 1% formic acid in water (eluent A) and acetonitrile (eluent B) by ultrasounding for deaeration prior to use. The flow rate was 1.0 mL/min at the following gradient program: 0.01–15 min, 7–11% B; 15–27 min, 11–26% B; 27–55 min, 26–27% B; 55–75 min, 27–40% B; 75–105 min, 40–100% B; 105–113 min, 100-7% B. The detection wavelength was set at 284 nm and an aliquot of 20 μL of sample solution was injected into the HPLC-DAD system. All samples and standards were filtered through a 0.22-μm Millipore membrane before use.

#### Standards preparation

The mixed standards solution was prepared by adding an accurately weighed amount of salvianolic acid B, danshensu, protocatechuate, protocatechualdehyde and rosmarinic acid to a volumetric flask and dissolved with 5 mL of methanol to make a final concentration of 500 µg/mL.

#### Sample preparation

An aliquot of 0.2 g of dried *S*. *miltiorrhizae* powder (filtered through a 50-mesh sieve) was accurately weighed and transferred into a 50-mL flask. After addition 20 mL of 5% methanol, the flask was weighed and transferred into an ultrasonic cleaning bath for extraction for 30 min at room temperature. Then, any lost weight was made up and the mixture was centrifuged at 10000 rpm for 10 minutes. Afterward, the supernatant was transferred into a centrifuge tube and filtered through a 0.22-μm Millipore membrane and the filtrate was collected as sample solution. All samples were stored at 4 °C in the dark until analysis.

#### Method validation

The methodology of chromatographic fingerprinting was validated for its precision, repeatability and stability. Intra- and inter-day variations were determined to assess the precision of the established HPLC-DAD method. Intra-day precision was evaluated by six successive injections of the randomly selected sample 27 (S27) solution on the same day, while inter-day precision was determined on five consecutive days. To confirm the repeatability, six different solutions prepared from S27 were analyzed. Meanwhile, the sample stability was examined by analysis of S27 solution stored at 4 °C for different time periods (0, 2, 4, 8, 24, 36 and 48 h). The variation for all the above assessment was expressed as relative standard deviation (RSD).

#### Similarity analysis

Under the optimized chromatographic conditions, each sample solution of 5% methanol extract from *S*. *miltiorrhizae* and the reference standard solution were injected into the HPLC system to create a HPLC-DAD chromatogram. Relevant information including peak area and retention time of all peaks was recorded and entered into the professional software named *Similarity Evaluation System for Chromatographic Fingerprint of Traditional Chinese Medicine* developed by Chinese Pharmacopoeia Committee (Version 2004A) (Beijing, China) to reconstruct the chemical fingerprints for automatic match. Similar chromatogram shapes generated from *S*. *miltiorrhizae* samples from different sources were difficult to identify or sort using only observation with the naked eye or microscopic observation. Therefore, hierarchical clustering analysis (HCA), a powerful chemometric method, was introduced.

#### Hierarchical clustering analysis

As a multivariate analysis technique, HCA can be used to sort samples into groups according to their internal characteristics. A dendrogram of all samples was obtained to represent the similarity or dissimilarity between samples. Each sample within the same group is similar to the others but different from those in other groups, with respect to a predetermined selection criterion. In this study, 32 batches of *S*. *miltiorrhizae* samples were analyzed using HCA according to the peak area of each of the 18 common peaks by HemI software (Heatmap Illustrator, version 1.0). Between-groups linkage clustering was chosen and the Euclidean distance was selected to assess the resemblance of the 32 samples, and then classify them into groups. Results were presented as a hierarchical cluster heat-map and two-way clustering was represented as a rectangular tiling of a data matrix, with clustering trees appended to the margins between characteristic peak areas. Within a relatively compact display area, this visual representation facilitates inspection of row, column, and joint cluster structure. This flexible architecture underscores the fact that a heat-map is a visual reaction of a statistical model. The hierarchical cluster heat-map is commonly used in the natural sciences and one of the most widely used graphs in the biological sciences.

### Microcalorimetric measurement

#### Sample preparation

An aliquot of 0.2 g dried *S*. *miltiorrhizae* powder (through a 50-mesh sieve) was accurately weighed and transferred into a 50-mL flask. After being added with 20-mL of 70% methanol, the flask was weighed and transferred into the ultrasonic cleaning bath for extraction for 30 min at room temperature. Then, the lost weight was made up and the mixture was centrifuged at 4000 rpm for 10 min. Afterward, the supernatant was transferred into a 125-mL crucible to dry on the water bath at 90 °C. All samples were stored at 4 °C in the dark. For the bioactive evaluation, the dried extract was re-dissolved in the LB culture medium to yield the final concentration of 1000 µg/mL.

#### Experimental procedure

First, all the 10 mL ampoules were washed with an acid detergent, rinsed with deionized water, and dried at 60 °C for 60 min in a drying cabinet, followed by autoclaving at 121 °C for 25 min. Initially, the concentration of *P*. *aeruginosa* inoculated into the LB culture medium was controlled at 2 × 10^6^ cells/mL. For the experiment, the first ampoule with no sample extract was regarded as the control, the remaining ampoules were aseptically filled with 6 mL of LB culture medium, 3 mL of *P*. *aeruginosa* suspension, and 1 mL of re-dissolved sample solution of *S*. *miltiorrhizae* from different sources. All experimental procedures were performed in a super-clean worktable. The ampoules were sealed with a crimped metal lid and silicon rubber seal. Ampules were then sequentially introduced into the instrument previously stabilized at 37 °C. Due to the temperature differences between the ampoules and the microcalorimeter, the ampoules will absorb heat. After about 45 min (when the temperature of ampoules reached 37 °C), the heat-flow power-time (*p*-*t*) curves were recorded until the recorder returned to baseline. All measurements were performed at 37 °C and all data were collected continuously using the dedicated software package.

#### Principal component analysis

Principal component analysis (PCA) is a simple, non-parametric method for extracting relevant information from a complex data set. It is a standard modern tool for data analysis used in various fields from neuroscience to computer graphics^28^. PCA was applied to reduce the dimensionality of the original data set and to explain the correlation of a large number of independent variables in terms of a smaller number of underlying factors (principal components or PCs) without losing much information^29^. From the power-time curve of *P*. *aeruginosa* growth in the presence of *S*. *miltiorrhizae*, many important parameters could be obtained for quantitative analysis. However, the different changing trends and occasional overlap of these parameters usually impedes quick and accurate explanation of the bioactivities of these samples on *P*. *aeruginosa*. To solve this problem, the main parameter(s) could be selected. Therefore, in this study, the quantitative thermo-kinetic parameters were analyzed by PCA using SIMCA-P 11.5 software (Umetrics AB, Umea, Sweden).

### Multiple linear regression analysis

Multiple linear regression analysis (MLRA) is usually used to model the best combination of two or more independent variables (*x*_i_) to predict or estimate the dependent variable (*Y*) by fitting a linear equation. It shows the contribution of each independent variable to the dependent variable in the following form:

4 $$Y={b}_{0}+\sum _{i=1}^{n}{b}_{i}{x}_{i}(n=1,2,3,4\ldots )$$

where *Y* is the estimated value and represents the dependent variable and *x*_i_ are the uncorrelated variables; *b*_0_ represents the estimated constant, and *b*_i_ is called the regression coefficients. In this study, MLRA was introduced to combine data from the chemical fingerprints (peak area of each component) and biothermal activity (main quantitative thermo-kinetic parameters) of *S*. *miltiorrhizae* samples on *P*. *aeruginosa*. This allowed us to establish the fingerprint-activity relationships using SPSS statistical software (SPSS for Windows 13.0, SPSS Inc., USA), to further explore the active components of *S*. *miltiorrhizae* that primarily contributed largely to the anti-*P*. *aeruginosa* activity.

## Electronic supplementary material

Supplementary Material
