# Supplementary material for: Combination of chemical fingerprint and bioactivity evaluation to explore the antibacterial components of Salvia miltiorrhizae
Source: Sci Rep. 2017 Aug 14;7:8112. doi: 10.1038/s41598-017-08377-0 (PMC5556045; doi:10.1038/s41598-017-08377-0)
Supplement: Supplementary file 1 — Supplementary Material [file 41598_2017_8377_MOESM1_ESM.pdf]

# Combination of chemical fingerprint and bioactivity evaluation to explore the antibacterial components of *Salvia miltiorrhizae*

Wei-Jun Kong*, Shan-Shan Zhang, Yan-Ling Zhao, Ming-Quan Wu, Ping Chen, Xiao-Ru Wu, Xin-Ping Ma, Wei-Ying Guo & Mei-Hua Yang*

**Table S1.** Peak areas of the common peaks from HPLC-DAD fingerprints of 32 *S. miltiorrhizae* samples.

| Samples | Peaks | | | | | | | | | | | | | | | | | |
|---|---|---|---|---|---|---|---|---|---|---|---|---|---|---|---|---|---|---|
| | 1 | 2 | 3 | 4 | 5 | 6 | 7 | 8 | 9 | 10 | 11 | 12 | 13 | 14 | 15 | 16 | 17 | 18 |
| S1 | 6943 | 18232 | 208836 | 10433 | 12871 | 893214 | 112714 | 180403 | 2693263 | 840185 | 317423 | 371139 | 3185152 | 2919102 | 41203 | 54302 | 41416 | 22700 |
| S2 | 27855 | 355436 | 61664 | 95052 | 163573 | 857715 | 68324 | 234870 | 1666050 | 585184 | 209062 | 33320 | 1863569 | 588798 | 16091 | 15891 | 27505 | 23488 |
| S3 | 3007 | 244149 | 16623 | 39995 | 248733 | 806148 | 57808 | 118603 | 1978997 | 664429 | 218377 | 101093 | 5684189 | 677458 | 11125 | 21118 | 29391 | 30265 |
| S4 | 6573 | 71730 | 12043 | 31607 | 2886 | 523274 | 46261 | 42372 | 316948 | 30562 | 51795 | 90045 | 412381 | 1237965 | 32972 | 17970 | 40286 | 43124 |
| S5 | 23545 | 48773 | 21513 | 13781 | 11927 | 166598 | 67864 | 4397 | 13341 | 26784 | 16829 | 9105 | - | 9316 | 28013 | - | 27253 | 44557 |
| S6 | 7666 | 44190 | 508307 | - | 159260 | 4080885 | 507875 | 458595 | 68282 | 69044 | 39365 | 173076 | - | 460530 | 30219 | 12057 | 26040 | 20107 |
| S7 | 19494 | 7125 | 53204 | - | 24796 | 202725 | 25233 | 32480 | 121621 | 75914 | 48101 | 19952 | 216568 | 89469 | 72596 | 76695 | 84642 | 84476 |
| S8 | 98416 | - | 18660 | - | 98702 | 675481 | 33320 | 125961 | 189579 | 79268 | 134783 | 247704 | 322270 | 11488 | 125886 | 60391 | 100565 | 56932 |
| S9 | 60490 | 119346 | 19494 | 48163 | - | 216861 | 8460 | 38171 | 35295 | 30868 | 40927 | 14800 | - | 9673 | 182010 | 65009 | 117401 | 78219 |
| S10 | 13483 | 111494 | - | 43320 | - | 198007 | 9887 | 37607 | 15556 | 36505 | 57000 | 7553 | - | 3860 | 83593 | 11467 | 67340 | 51360 |
| S11 | 100908 | 925197 | 687386 | 97888 | 831329 | 400921 | 57985 | - | 1089429 | 121429 | 144321 | 2336699 | 29727606 | 813255 | 64737 | 26138 | 61583 | 28995 |
| S12 | 162872 | - | - | - | 553369 | 233834 | 78833 | - | 1953942 | 3117684 | - | 2694280 | 44617821 | - | 59879 | 167928 | 50034 | 11194 |
| S13 | 96135 | 397245 | 484954 | 294605 | 237317 | 2073021 | 40545 | 68183 | 2821196 | 166119 | 328384 | 92405 | 2351493 | 1122463 | 106349 | 53539 | 110696 | 105460 |
| S14 | 196509 | 84427 | 602121 | - | 719769 | 273967 | 72217 | - | 264237 | 1203627 | - | 676602 | 41503420 | 94821 | 41311 | 36694 | 28576 | 27874 |
| S15 | 82090 | 22257 | 75611 | - | 478071 | 221448 | 19081 | 2480402 | 780947 | 1805917 | - | 1221771 | 295868 | - | 66363 | 16020 | 20835 | 7274 |
| S16 | - | 33391 | - | - | 32933 | 719773 | 117381 | 223798 | 980703 | 1198462 | 568216 | 217034 | 5245173 | 769689 | 99221 | 96774 | 89623 | 33551 |
| S17 | 61896 | 131249 | 17642 | 97831 | 234385 | 981203 | 220052 | 249432 | 524267 | 1147671 | 522963 | 81843 | 1559076 | 499645 | 181928 | 166947 | 246836 | 166315 |
| S18 | 22942 | - | 44578 | - | 19599 | 848215 | 319263 | 345069 | 1386966 | 1123252 | 531642 | 483192 | 5884111 | 1303076 | 186059 | 33725 | 173008 | 139860 |
| S19 | 70897 | 116310 | 40743 | 97124 | 307662 | 741906 | 293868 | 219227 | 901508 | 1387897 | 573364 | 127207 | 3106705 | 532683 | 70302 | 52304 | 70064 | 51558 |
| S20 | 69688 | - | - | - | 67735 | 929240 | 262665 | 265975 | 489658 | 102425 | 134569 | - | - | 432190 | 153272 | 55918 | 81507 | 57514 |
| S21 | 39686 | 107377 | 15707 | 113606 | 122147 | 809181 | 277783 | 225288 | 905151 | 317201 | 370082 | - | - | 338558 | 136479 | 114510 | 172388 | 128754 |
| S22 | 71624 | 99854 | 60771 | - | 384829 | 774964 | 489236 | 319042 | 995271 | 1388205 | 736947 | 440398 | 5449876 | 238013 | 106694 | 179202 | 192193 | 78524 |
| S23 | 129780 | 167066 | 252800 | 150243 | 447436 | 1355312 | 307709 | 359415 | 1736214 | 1742480 | 628515 | 371813 | 5060853 | 415243 | 108625 | 55094 | 65674 | 30214 |

| | | | | | | | | | | | | | | | | | | |
|---|---|---|---|---|---|---|---|---|---|---|---|---|---|---|---|---|---|---|
| S24 | 11838 | 17762 | - | - | 32965 | 1019893 | 313390 | 230565 | 176952 | 1394385 | 405248 | 619431 | 7480813 | 298906 | 89482 | 52873 | 56328 | 15504 |
| S25 | 86348 | - | 264677 | - | 523606 | 1279685 | 181287 | 318120 | 1390471 | 1160513 | 582947 | 100969 | 4483297 | 389443 | 36727 | 58078 | 63593 | 29706 |
| S26 | 21455 | 65293 | 167793 | - | 448342 | 472505 | 252686 | 332329 | 1953707 | 1447125 | 678588 | 762970 | 10875604 | 404310 | 134252 | 139381 | 120224 | 41054 |
| S27 | 10819 | 24045 | 114479 | - | - | 271473 | 122203 | 274520 | 1013411 | 1072161 | 470075 | 1674395 | 16610886 | 494316 | 87544 | 20095 | 69684 | 30385 |
| S28 | - | 104171 | - | - | 473068 | 398142 | 324121 | 281411 | 1612514 | 906465 | 443513 | 1166596 | 18801720 | 320871 | 85500 | 83265 | 82079 | 24327 |
| S29 | 14498 | 12379 | 923307 | - | - | 1118212 | 104590 | 164574 | 4690527 | 829802 | 199439 | 1983480 | 2208331 | 1063340 | 25920 | 58338 | 25180 | 37165 |
| S30 | 6837 | 39517 | 179397 | - | 309047 | 1389845 | 131650 | 606155 | 1147532 | 1577144 | 757197 | 182344 | 1448101 | 686731 | 110786 | 63462 | 97185 | 76334 |
| S31 | 17991 | 22010 | 172862 | - | 31204 | 266799 | 86412 | 160835 | 1352778 | 851582 | 327789 | 1312088 | 19214638 | 907744 | 104561 | 25438 | 78615 | 20820 |
| S32 | 7943 | 7432 | - | - | 52070 | 418440 | 36718 | 69551 | 1012770 | 359774 | 100000 | 691589 | 6306373 | 559245 | 102678 | 39480 | 68900 | 22759 |
| C.V.[a] (%) | 102.8 | 67.0 | 81.1 | 115.7 | 107.3 | 107.7 | 114.3 | 65.9 | 112.9 | 117.4 | 140.7 | 82.9 | 76.8 | 103.5 | 178.0 | 132.5 | 151.4 | 130.5 |

[a] C.V.% = $\sigma/\mu \times 100$; σ was the standard deviation, μ was the average value of the peak area.

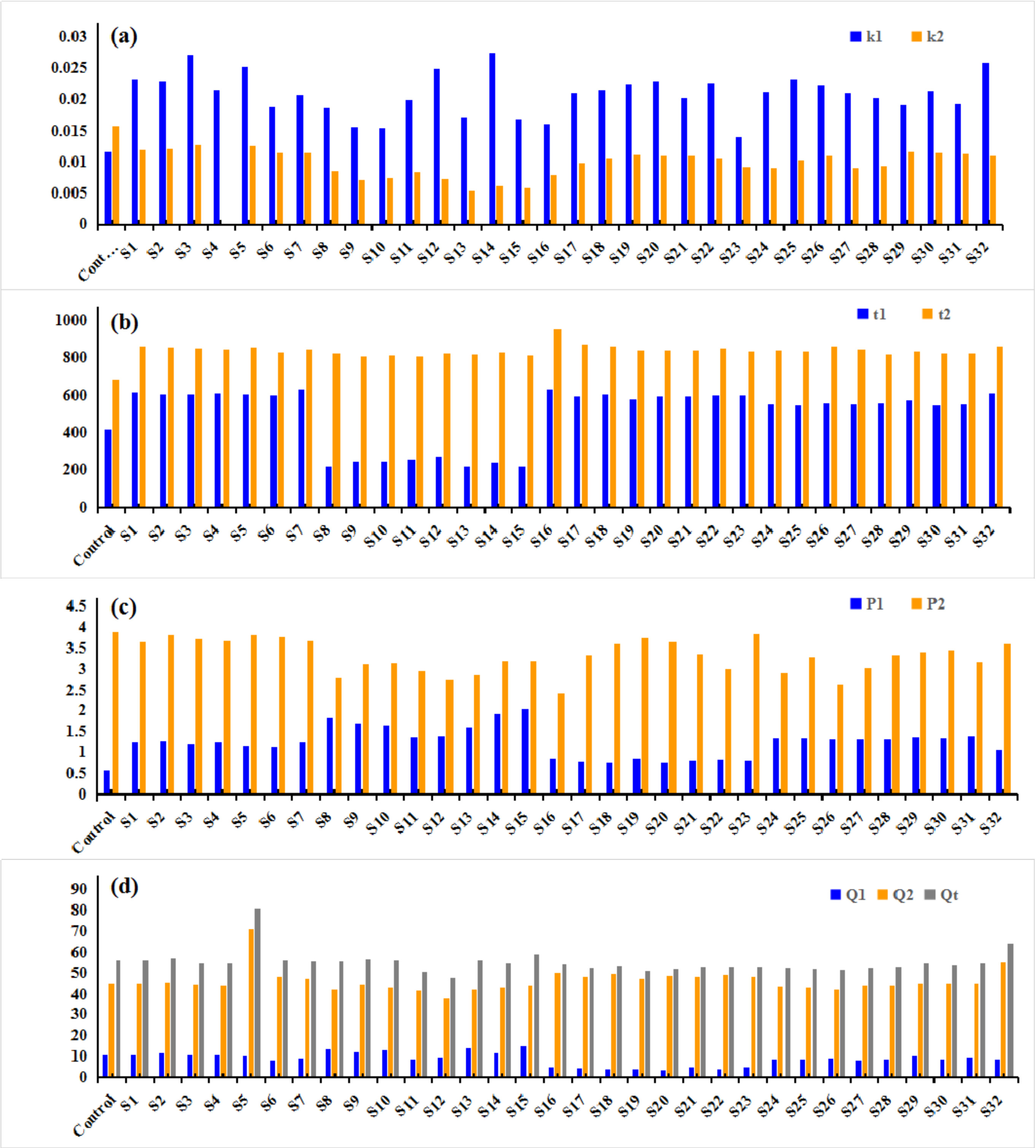


**Figure S1.** Thermokinetic parameters *P. aeruginosa* growth affected by 32 *S. Miltiorrhizae* samples from different sources. (a) Growth rate constant $k_1$ and $k_2$; (b) peak value of highest peak $P^1_m$ and $P^2_m$; (c) appearance time of the highest peak $t^1_m$ and $t^2_m$; (d) heat output of the two stages $Q_1$, $Q_2$, and the total heat output $Q_t$.
